# What Physiotherapists Specialized in Orthopedic Manual Therapy Know About Nocebo-Related Effects and Contextual Factors: Findings From a National Survey

**DOI:** 10.3389/fpsyg.2020.582174

**Published:** 2020-10-20

**Authors:** Giacomo Rossettini, Tommaso Geri, Alvisa Palese, Chiara Marzaro, Mattia Mirandola, Luana Colloca, Mirta Fiorio, Andrea Turolla, Mattia Manoni, Marco Testa

**Affiliations:** ^1^Department of Neuroscience, Rehabilitation, Ophthalmology, Genetics, Maternal and Child Health, University of Genova, Genova, Italy; ^2^School of Physiotherapy, University of Verona, Verona, Italy; ^3^Department of Medical Sciences, University of Udine, Udine, Italy; ^4^Department of Pain and Translational Symptom Science, School of Nursing, University of Maryland, Baltimore, Baltimore, MD, United States; ^5^Department of Anesthesiology and Psychiatry, School of Medicine, Center to Advance Chronic Pain Research, University of Maryland, Baltimore, Baltimore, MD, United States; ^6^Department of Neurosciences, Biomedicine and Movement Sciences, University of Verona, Verona, Italy; ^7^Department of Neurorehabilitation Technologies, San Camillo IRCCS srl, Venezia, Italy

**Keywords:** nocebo effect, expectation, physiotherapy (MeSH), contextual factors, pain, placebo effects, conditioning, survey

## Abstract

**Objective:**

The aim of this study was to investigate the knowledge of orthopedic manual therapists (OMTs) regarding context factors (CFs) capable of triggering nocebo effects during the treatment and how this knowledge is related to their socio-demographic features.

**Design:**

A cross-sectional online survey.

**Setting:**

National.

**Main Outcome Measures:**

A 20 items questionnaire composed by open-ended and closed single-choice questions was administered to explore: (a) socio-demographic variables (10 questions); (b) the relation between different CFs and nocebo-related effects (2 questions); and (c) the knowledge of participants about nocebo-related effects and how they managed them in the clinical practice (8 questions).

**Participants:**

1288 OMTs were recruited from the database of the Master in Rehabilitation of Musculoskeletal Disorders (MRDM) of the University of Genova from March to May 2019. Inclusion criteria were: (a) to possess a valid email account; (b) to understand and use as a native language the Italian; (c) to be graduated as OMTs; and (d) to be employed as physiotherapists specialized-OMTs during the survey.

**Results:**

791 responses were received (61.4%); 473 of them were male (59.8%), with an average age of 31.0 ± 7.1 years. OMTs defined nocebo-related effects as the psychosocial context effects around therapy and patient with specific biological bases (72.2%). OMTs know that their clinical practice is pervaded by nocebo-related effects (42.5%), triggered by CFs. Participants communicated nocebo-related effects balancing the positive features of the therapy with the negative ones (50.9%), during the decision of the therapeutic plan (42.7%). They reported associative learning as the main mechanism involved in nocebo-related effects (28.8%). OMTs taught and trained patient’s strategies to manage nocebo-related effects (39.6%) through an evaluation and correction of patient’s anxieties, doubts and expectations (37.7%). OMTs most frequently considered themselves to have a “medium” education about nocebo-related effects (48.2%) and that their management should be taught during bachelor (78.6%).

**Conclusion:**

OMTs believed that nocebo-related effects were present in their clinical practice and that they can be triggered by CFs.

## Introduction

Placebo and nocebo-related effects are emerging phenomena of interest among researchers, scholars and clinicians in orthopedic manual therapy (Rossettini et al., 2018a). They represent the result of the positive (placebo) or negative (nocebo) use of contextual factors (CFs) during the administration of a therapy (Benedetti, 2013). Contextual factors include physical, psychological and social elements involved in the clinical encounter between the patient and the physiotherapist (Di Blasi et al., 2001) such as: (a) physiotherapist’s features (e.g., expertise, reputation); (b) patient’s features (e.g., expectations, previous experience); (c) patient-physiotherapist relationship (e.g., verbal communication, posture); (d) treatment features (e.g., overt therapy, marketing); and (e) healthcare setting features (e.g., environment, architecture) (Testa and Rossettini, 2016). In the clinical scenario, the interaction between the specific component of a therapy and the surrounding CFs influences the subjective therapeutic experience (e.g., pain, fear, anxiety) triggering placebo or nocebo-related effects (Carlino et al., 2014): specifically, positive CFs can ameliorate the clinical outcomes, while negative CFs can amplify patients’ symptoms preventing their recovery (Wager and Atlas, 2015).

While placebo-related effects have been widely inquired in orthopedic manual therapy, nocebo-related effects have been underlined as a new research field that should be investigated for several reasons (Rossettini et al., 2020). First, psychobiological explanations have been documented as the underlying mechanisms of action (e.g., genetic, expectation, learning) of CFs and evoked nocebo-related effects (Colloca and Barsky, 2020) capable to exacerbate the perception of a symptom affecting also the therapeutic relationship (Hansen and Zech, 2019). Second, specific neurotransmitters (e.g., cholecystokinin and cyclooxygenase-prostaglandins activation; opioid and dopamine deactivation) have been indicated as mediators involved in CFs and triggered nocebo-related effects (Frisaldi et al., 2015). Part of these processes are also the activation of neural pathways (e.g., anterior cingulate cortex, dorsolateral prefrontal cortex, periaqueductal gray, and spinal cord) (Darnall and Colloca, 2018). Third, the negative clinical impact of CFs (e.g., patients’ expectations, beliefs) and induced nocebo-related effects on therapeutic outcomes has been highlighted at multiple healthcare levels, resulting in increased costs, work absenteeism and medicalization (Hallegraeff et al., 2012; Trinderup et al., 2018).

At the international level, an expert panel has recently identified as a research priority the knowledge nocebo-related effects and CFs among clinicians (Evers et al., 2018). To date, one qualitative study has investigated nocebo-related effects during the physician-patient communication in Pakistan (Ashraf and Saaiq, 2014); while two Italian surveys have explored the knowledge of CFs and placebo-related effects including physiotherapists specialized in orthopedic manual therapy and nurses (Rossettini et al., 2018b; Palese et al., 2019), thus leaving still unexplored this research field. In particular, OMTs represent an ideal group of clinicians to be investigated because their practice is intrinsically pervaded by CFs: during the administration of each therapy (e.g., joint mobilization, massage, exercise) they use CFs (e.g., verbal and non-verbal communication) influencing the outcome (Rossettini et al., 2018a).

Thus, the aim of this study was to investigate the knowledge of orthopedic manual therapists (OMTs) regarding context factors (CFs) capable of triggering nocebo effects during the treatment and how this knowledge is related to their socio-demographic features.

## Materials and Methods

### Design

This quantitative cross-sectional survey was conducted according to the Checklist for Reporting Results of Internet E-Surveys (CHERRIES) guidelines (Eysenbach, 2004) and the STrengthening the Reporting of OBservational Studies in Epidemiology (STROBE; Von Elm et al., 2007), from March to May 2019. All the procedures were approved by the Liguria Clinical Experimental Ethics Committee (P.R.236REG2016, accepted on 19/07/2016).

### Participants and Settings

Participants were Italian physiotherapists specialized and graduated as OMTs (Rossettini et al., 2018b). Our sample of OMTs was recruited from the database of the Master in Rehabilitation of Musculoskeletal Disorders of Genova University (*n* = 1288). This higher educational program represents approximately the totality of the Italian physiotherapists specialized as OMT. Furthermore, it is the oldest academic post-graduate program in manual therapy in Italy (Bologna Working Group, 2005) based upon the standards established by the International Federation of Orthopedic Manipulative Physical Therapists (IFOMPT, 2016).

Inclusion criteria were: (a) to possess a valid email account; (b) to understand and use as a native language the Italian; (c) to be graduated as OMTs; and (d) to be employed as physiotherapists specialized-OMTs during the survey. Exclusion criteria were: (a) to possess an invalid email account; (b) to use and understand different languages than Italian; (c) to be trained as OMT student during the survey; and (d) to be employed as non-specialized physiotherapists.

From the total population target of 1288 OMTs, approximately 516–773 responses were expected, based on previous studies placebo-related effects and CFs in which the response rate was from 30 to 60% (Rossettini et al., 2018b; Palese et al., 2019). The application of these predicted values to the formula for estimating the sample size using a single population proportion with the population proportion set at 50.0% produced a two−sided 95.0% confidence level of 2.2–3.3% points of the true value and a relative standard error ranging from 2.3 to 3.4 (Australian Bureau of Statistics, 2020).

### Questionnaire Development and Pre-testing

The questionnaire adopted in this study was adapted from a previous survey published on CFs and placebo-related effects among OMTs (Rossettini et al., 2018b). Using distinct and iterative steps, a panel of six experts in nocebo-related effects, CFs and survey design: (a) modified the meaning of items from a positive (=placebo) to a negative (=nocebo) meaning; (b) evaluated items for face and content validity of the new version of the questionnaire; and (c) valued the content accuracy, survey structure and word clarity (De Leeuw et al., 2008). In a first phase each member of the panel worked independently; in a second phase they discussed and confronted using a thinking aloud strategy. The final survey tool was composed by 20 items available in Italian (Supplementary File 1) and English (Supplementary File 2).

The survey was piloted as a self-administered questionnaire in a convenient sample of 15 OMTs (North, *n* = 5; Center, *n* = 5; South of Italy, *n* = 5), not included in final sample of the study. To investigate potential filled in issues (e.g., vague questions, unclear words), a telephone debriefing session (De Leeuw et al., 2008) among the involved 15 OMTs was performed. The outcome of the pilot study was positive: the sample referred that the questions did not need further explanation, and the words were simple and easy to understand: thus, no changes to the items were applied.

### Questionnaire Implementation

The self-administered questionnaire was divided into three sections (A, B, and C). In the first section (A) the socio-demographic variables were collected using two open-ended questions (age, years of clinical practice) and eight closed single-choice questions (gender, Italian region, workplace, type of work, setting, profile of patients cared for, field of work, hours of work per week).

The section (B) included variables exploring the relation between different CFs and nocebo-related effects by using two closed single-choice questions. Specifically, the questions investigated the frequency of nocebo-related effects in the OMTs’ experience (answers from “never” to “always”) and the beliefs about the weight of specific CFs (Likert from 0 “not at all” to 4 “a lot of”) in triggering nocebo-related effects.

The last section (C) included variables exploring the knowledge of participants about nocebo-related effects and how they managed them in the clinical practice. In particular, eight closed single-choice questions investigated: (a) the communication (*n* = 2); (b) the mechanisms of action (*n* = 1); (c) the management issues (*n* = 2); (d) the education (*n* = 2); and (e) the definition (*n* = 1) of CFs and nocebo-related effects.

### Data Collection Procedure

The SurveyMonkey (Survey-Monkey, Palo Alto, California)^1^ was selected as an online survey tool. Orthopedic manual therapists were invited to participate in the study through an email (De Leeuw et al., 2008) in which the aim of the study and the anonymity of data were explained. The email also pointed that the informed consent to participate in the study would have been provided by clicking on the survey link (Eysenbach and Wyatt, 2002). Participants could change their answers and review them before submitting the final survey, but they were required to answer all questions to prevent missing data. The survey took responders between 5 and 10 minutes to complete: this response time was chosen to optimize responses rate (Fan and Yan, 2010).

Data were downloaded and stored in an encrypted computer. Only the principal investigator could have access to the information achieved during all stages of the study. All data (name and email address) were anonymized to ensure privacy and data protection (De Leeuw et al., 2008) leaving the participants’ identities concealed to researchers (Eysenbach and Wyatt, 2002). No incentives were offered to participants and the attendance was voluntary (Eysenbach and Wyatt, 2002). The OMTs who did not complete the questionnaire were encouraged to participate in the survey by an email reminder at 2, 4, and 8 weeks after the first contact.

### Data Analysis

To review answers accuracy, data were transferred from SurveyMonkey to Excel spreadsheet. For descriptive statistic, continuous variables were reported using mean, standard deviation (SD) and confidence intervals at 95% (95% CI); whereas absolute frequency and percentage described dichotomous, nominal and ordinal variables, coming from single answer questions. Age and years of clinical practice were transformed into ordinal variables considering a decade as variable level for the analysis of correlations.

As this study is the first on nocebo in physiotherapy, we analyzed all the possible relations between the socio-demographic characteristics of the sample (section A, intended as the dependent variables) and answers to section (B) and (C), intended as independent variables, were analyzed with Cramer’s V, which is a statistic analysis tool that measures strength and directions of associations used when one or both the independent and dependent variables consists of unordered categories with more than two levels. Only correlation values higher than the threshold (>0.50) (Cohen, 1988) were accepted and reported in the study. R software was used for the data analysis (R Core Team, 2020) with the packages psych (Revelle, 2017) and ggplot2 (Wickham, 2009).

## Results

### Flow of Participants Through the Study

From the sample of 1288 OMTs, 791 responses were received (61.4%). Most participants were male (*n* = 473; 59.8%; 95% CI 56.3–63.2) and their average age was 31.0 ± 7.1 years. Overall, 70.9% of OMTs (*n* = 561; 95% CI 67.6–74.0) reported to work in the North of Italy. Respondents had an average clinical experience of 7.4 ± 6.3 years. The majority of them was employed in private health care settings (*n* = 676; 85.5%; 95% CI 82.8–87.8) as a freelance professional (*n* = 569; 71.9%; 95% CI 68.6–75.0) working between 32 and 45 h per week (*n* = 433; 54.7%; 95% CI 51.2–58.2) in an outpatient clinic (*n* = 607; 76.7%; 95% CI 73.6–79.6). A high proportion of OMTs worked in the musculoskeletal field (*n* = 718; 90.8%; 95% CI 88.5–92.7) with adult patients (646; 81.7%; 95% CI 78.8–84.3). The participants’ demographics are described in Table 1.

**TABLE 1:** Socio-demographic variables.

Demography	Values	95% CI
***Years, mean (SD)***	31.0 (7.1)	30.5–31.5
***Years of clinical practice, mean (SD)***	7.4 (6.3)	7.0–7.8
***Gender, n (%)***		
Male	473 (59.8)	56.3–63.2
Female	318 (40.2)	36.8–43.7
***Italian region, n (%)***		
North	561 (70.9)	67.6–74.0
Center	161 (20.4)	17.6–23.4
South	69 (8.7)	6.9–11.0
***Workplace, n (%)***		
Private health care settings	676 (85.5)	82.8-87.8
Public health care settings	115 (14.5)	12.2-17.2
***Type of work, n (%)***		
Freelance professional	569 (71.9)	68.6–75.0
Employee	222 (28.1)	25.0–31.4
***Setting, n (%)***		
Outpatient clinic	607 (76.7)	73.6–79.6
Hospital	123 (15.5)	13.1–18.3
Residential care (nursing home)	61 (7.7)	6.0–9.9
***Profile of patients, n (%)***		
Adults	646 (81.7)	78.8–84.3
Older people	134 (16.9)	14.4–19.8
Pediatrics	11 (1.4)	0.7–2.6
***Field of work, n (%)***		
Musculoskeletal	718 (90.8)	88.5–92.7
Neurological	57 (7.2)	5.5–9.3
Cardiorespiratory	11 (1.4)	0.7–2.6
Oncological	3 (0.4)	0.1–1.2
Uro-gynecological	2 (0.3)	0.0–1.0
***Hours of work per week, n (%)***		
1–15	18 (2.3)	1.4–3.6
16–30	175 (22.1)	19.3–25.2
31–45	433 (54.7)	51.2–58.2
46–60	146 (18.5)	15.8–21.4
>60	19 (2.4)	1.5–3.8

**n*, number of participants;%, percentage; SD, standard deviation; 95% CI, 95% confidence interval; >, more.*

### Definition of Nocebo-Related Effects

Orthopedic manual therapists were asked how they would define nocebo-related effects: the most selected option was “psychosocial effects of the context around therapy and patient with specific biological bases” (*n* = 571; 72.2%; 95% CI 68.9–75.3). Some OMTs opted for “health procedure effects able to create negative expectations” (*n* = 162; 20.5%; 95% CI 17.7–23.5), whereas the less frequent response was “adverse responses observed in people of the control group of randomized clinical trials” (*n* = 58; 7.3%; 95% CI 5.7–9.4).

### Mechanisms of Action of Nocebo-Related Effects

Analyzing the mechanisms of action that are believed to explain nocebo-related effects, the most frequent response was “associative learning (e.g., conditioning)” (*n* = 228; 28.8%; 95% CI 25.7–32.1), followed by “patient’s expectation” (*n* = 207; 26.2%; 95% CI 23.2–29.4). Other options were (in descending order): “psychological traits” (*n* = 125; 15.8%; 95% CI 13.4–18.6), “previous experiences” (*n* = 95; 12.0%; 95% CI 9.9–14.5), “social learning” (*n* = 66; 8.3%; 95% CI 6.6–10.5), and “neurophysiological” mechanisms (*n* = 52; 6.6%; 95% CI 5.0–8.6). The less chosen option regarding the mechanism of action was “genetic” (*n* = 18; 2.3%; 95% CI 1.4–3.6).

### Beliefs About CFs as Triggers of Nocebo-Related Effects

Participants reported a high level of conviction toward CFs (mean = 2.4 out of 4; 95% CI 2.4–2.5) as triggers of nocebo-related effects. Specifically, the most important CFs were (in descending order): “lack of empathetic therapeutic alliance with the patient” (mean = 3.3; 95% CI 3.3–3.4), “patient’s negative expectation” (mean = 3.3; 95% CI 3.2–3.4), “patient’s previous negative experience” (mean = 3.2; 95% CI 3.1–3.2); “negative verbal communication” (mean = 3.1; 95% CI 3.0-3.1); and “negative attitudes and pessimistic behavior” (mean = 3.1; 95% CI 3.0-3.1).

Less influent CFs were (in descending order): “printed information about the therapy” (mean = 1.9; 95% CI 1.8–1.9); “hidden therapy” (mean = 1.8; 95% CI 1.7–1.8); “inaccurate design” (mean = 1.7; 95% CI 1.7–1.8), “lack of patient’s familiarity with the therapy” (mean = 1.6; 95% CI 1.6–1.7); “lack of uniform” (mean = 1.6; 95% CI 1.5–1.7). A complete description of CFs capable to trigger nocebo-related effects is reported in Table 2.

**TABLE 2:** Beliefs about CFs as triggers of nocebo-related effects.

	Likert score mean (95% CI)	4 n (%); 95% CI	3 n (%); 95% CI	2 n (%); 95% CI	1 n (%); 95% CI	0 n (%); 95% CI
**A: *Weak professional reputation*** (e.g., qualification, expertise of physiotherapist)	2.6 (2.6–2.7)	136 (17.2); 14.7–20.0	360 (45.5); 42.0–49.1	207 (26.2); 23.2–29.4	47 (5.9); 4.4–7.9	41 (5.2); 3.8–7.0
**A. *Lack of uniform*** (e.g., white coat of physiotherapist)	1.6 (1.5–1.7)	15 (1.9); 1.1–3.2	116 (14.7); 12.3–17.4	284 (35.9); 32.6–39.4	296 (37.4); 34.1–40.9	80 (10.1); 8.1–12.5
**A. *Negative attitudes and pessimistic behavior*** (e.g., toward a patient’s dysfunctions)	3.1 (3.0–3.1)	279 (35.3); 32.0–38.7	373 (47.1); 43.6–50.7	87 (11.0); 8.9–13.4	19 (2.4); 1.5–3.8	33 (4.2); 2.9–5.9
**B. *Patient’s negative expectation*** (e.g., toward a physiotherapy treatment)	3.3 (3.2–3.4)	426 (53.9); 50.3–57.4	271 (34.3); 31.0–37.7	42 (5.3); 3.9–7.2	18 (2.3); 1.4–3.6	34 (4.3); 3.0–6.0
**B. *Patient’s previous negative experience*** (e.g., toward a physiotherapy treatment)	3.2 (3.1–3.2)	343 (43.4); 39.9–46.9	314 (39.7); 36.3–43.2	89 (11.2); 9.2–13.7	5 (0.6); 0.2–1.6	40 (5.1); 3.7–6.9
**C. *Negative verbal*** *communication* (e.g., medical language, lack of positive messages associated with the treatment)	3.1 (3.0–3.1)	298 (37.7); 34.3–41.2	334 (42.2); 38.8–45.8	98 (12.4); 10.2–14.9	24 (3.0); 2.0–4.5	37 (4.7); 3.4–6.5
**C. *Negative non-verbal communication*** (e.g., closing posture, gestures, absence of eye contact, facial expressions)	2.7 (2.6–2.8)	167 (21.1); 18.3–24.2	345 (43.6); 40.1–47.2	197 (24.9); 22.0–28.1	41 (5.2); 3.8–7.0	41 (5.2); 3.8–7.0
**C. *Lack of empathetic therapeutic alliance with the patient*** (e.g., lack of active listening)	3.3 (3.3–3.4)	373 (47.1); 43.6–50.7	316 (39.9); 36.5–43.5	90 (11.4); 9.3–13.8	11 (1.4); 0.7–2.5	1 (0.1); 0.0–0.8
**D. *Information about the therapy delivered by other patients*** (e.g., negative communicated or observed responses)	2.5 (2.4–2.5)	95 (12.0); 9.9–14.5	324 (41.0); 37.5–44.5	267 (33.7); 30.5–37.2	68 (8.6); 6.8–10.8	37 (4.7); 3.4–6.4
**D. *Printed information about the therapy*** (e.g., medical leaflets)	1.9 (1.9–2.0)	37 (4.7); 3.4–6.4	187 (23.6); 20.7–26.8	319 (40.3); 36.9–43.8	196 (24.8); 21.8–28.0	52 (6.6); 5.0–8.6
**D. *Information about the therapy from the media*** (e.g., internet, social media, television news)	2.5 (2.5–2.6)	158 (20.0); 17.3–23.0	291 (36.8); 33.4–40.3	216 (27.3); 24.3–30.6	83 (10.5); 8.5–12.9	43 (5.4); 4.0–7.3
**D. *Hidden therapy*** (e.g., impossibility for the patient to see when the therapy is delivered)	1.8 (1.7–1.8)	27 (3.4); 2.3–5.0	150 (19.0); 16.3–21.9	300 (37.9); 34.5–41.4	235 (29.7); 26.6–33.0	79 (10.0); 8.0–12.3
**D. *Sudden interruption of the therapy*** (e.g., to attend other patients or colleagues)	2.3 (2.2–2.4)	38 (4.8); 3.5–6.6	287 (36.3); 32.9–39.8	346 (43.7); 40.3–47.3	110 (13.9); 11.6–16.6	10 (1.3); 0.6–2.4
**D. *Marketing of the therapy*** (e.g., cost, brand, color, shape)	2.1 (2.0–2.2)	49 (6.2); 4.7–8.2	233 (29.5); 26.3–32.8	314 (39.7); 36.3–43.2	144 (18.2); 15.6–21.1	51 (6.4); 4.9–8.4
**D. *Lack of patient’s familiarity with the therapy*** (e.g., new therapy)	1.6 (1.6–1.7)	18 (2.3); 1.4–3.6	121 (15.3); 12.9–18.0	281 (35.5); 32.2–39.0	301 (38.0); 34.7–41.5	70 (8.8); 7.0–11.1
**D. *Lack of patient-centered approach*** (e.g., not shared-decision of physiotherapy treatment)	2.6 (2.5–2.7)	148 (18.7); 16.1–21.6	317 (40.1); 36.7–43.6	217 (27.4); 24.4–30.7	73 (9.2); 7.3–11.5	36 (4.5); 3.2–6.3
**D. *Inappropriate physical contact with the patient*** (e.g., invasiveness of the touch)	2.6 (2.5–2.7)	152 (19.2); 16.6–22.2	334 (42.2); 38.8–45.8	204 (25.8); 22.8–29.0	58 (7.3); 5.7–9.4	43 (5.4); 4.0–7.3
**E. *Lack of comfortable setting*** (e.g., inappropriate lighting, temperature)	2.4 (2.4–2.5)	96 (12.1); 10.0–14.7	321 (40.6); 37.1–44.1	242 (30.6); 27.4–34.0	92 (11.6); 9.5–14.1	40 (5.1); 3.7–6.9
**E. *Inadequate environmental architecture*** (e.g., inappropriate highlights, indicators)	2.0 (1.9–2.1)	35 (4.4); 3.1–6.2	223 (28.2); 25.1–31.5	293 (37.0); 33.7–40.5	187 (23.6); 20.7–26.8	53 (6.7); 5.1–8.7
**E. *Inaccurate design*** (e.g., absence of decorations, ornaments, colors)	1.7 (1.7–1.8)	18 (2.3); 1.4–3.6	151 (19.1); 16.4–22.0	293 (37.0); 33.7–40.5	258 (32.6); 29.4–36.0	71 (9.0); 7.1–11.2

**n*, number of participants; 95% CI, 95% confidence interval; 0, not at all; 1, few; 2, enough; 3, much; 4, a lot of; A, physical therapist domain; B, patient domain; C, physical therapist-patient relationship domain; D, therapy domain; E, healthcare setting domain.*

### Frequency of Nocebo-Related Effects

Orthopedic manual therapists reported that nocebo-related effects were present in their clinical experience with a frequency of (in descending order): “sometimes” (*n* = 336; 42.5%; 95% CI 39.0–46.0), “often” (*n* = 286; 36.2%; 95% CI 32.8–39.6), “rarely” (*n* = 147; 18.6%; 95% CI 16.0–21.5), “always” (*n* = 11; 1.4%; 95% CI 0.7–2.5) and “never” (*n* = 11; 1.4%; 95% CI 0.7–2.5).

### Communication of Nocebo-Related Effects

When asked how participants were used to communicate nocebo-related effects to the patient, the most frequent answer was “balance the positive features of the therapy with the negative ones” (*n* = 403; 50.9%; 95% CI 47.4–54.5), whereas few OMTs reported to “do not say anything” (*n* = 77; 9.7%; 95% CI 7.8–12.1).

Regarding when they communicate nocebo-related effects, most of OMTs informed their patients “during the decision of the therapeutic plan” (*n* = 338; 42.7%; 95% CI 39.3–46.3). The option “during the clinical examination” (*n* = 17; 2.1%; 95% CI 1.3–3.5) was the less chosen. The detailed communication strategies used in daily practice are reported in Table 3.

**TABLE 3:** Communication of nocebo-related effects.

Communication *n* (%)	Values	95% CI
***How do you mainly communicate nocebo effects to the patient?***		
Balance the positive features of the therapy with the negative ones	403 (50.9)	47.4–54.5
Carefully explain the effects and the role played by the negative context	226 (28.6)	25.5–31.9
Minimize negative information on nocebo-related effects by not reporting all the elements	85 (10.7)	8.7–13.2
Do not say anything	77 (9.7)	7.8–12.1
***When do you mainly communicate nocebo effects to the patient?***		
During the decision of the therapeutic plan	338 (42.7)	39.3–46.3
During the administration of the therapy	221 (27.9)	24.9–31.2
Do not communicate anything	114 (14.4)	12.1–17.1
During the anamnesis	56 (7.1)	5.4–9.1
During the formulation of the diagnosis	45 (5.7)	4.2–7.6
During the clinical examination	17 (2.1)	1.3–3.5

**n*, number of participants;%, percentage; 95% CI, 95% confidence interval.*

### Management of Nocebo-Related Effects

The most adopted intervention to avoid nocebo-related effects was “teach and train patient’s strategies to manage adverse events” (*n* = 313; 39.6%; 95% CI 36.2–43.1). The less chosen responses were “refer to evidence-based information on the Internet” (*n* = 14; 1.8%; 95% CI 1.0–3.0) and “adopt a gradual reduction of the treatment in a hidden way” (*n* = 9; 1.1%; 95% CI 0.6–2.2).

When asked which clinician-patient communication was mainly adopted to avoid nocebo-related effects, the majority of OMTs replied “evaluate and modify patient’s anxieties, doubts and expectations” (n = 298; 37.7%; 95% CI 34.3–41.2), whereas a minor number of OMTs chose “ask the patient to give questions” (*n* = 17; 2.1%; 95% CI 1.3–3.5). Table 4 presented the overall responses about the management of nocebo-related effects.

**TABLE 4:** Management of nocebo-related effects.

Management of nocebo-related effects, *n* (%)	Values	95% CI
***Which interventions do you mainly use to avoid nocebo effects?***		
Teach and train patient’s strategies to manage nocebo-related effects	313 (39.6)	36.2–43.1
Optimize expectations toward treatment and nocebo-related effects	196 (24.8)	21.8–28.0
Explain nocebo-related effects using illustrative methods (e.g., videos, figures, graphs and percentages) and simple language	110 (13.9)	11.6–16.6
Present first the positive features of the treatment and then the negative ones	71 (9.0)	7.1–11.2
Do not do anything	46 (5.8)	4.3–7.7
Use pre-treatments with a reduced percentage of nocebo-related effects (e.g., active or inert treatment-test)	32 (4.0)	2.8–5.7
Refer to evidence-based information on the Internet	14 (1.8)	1.0–3.0
Adopt a gradual reduction of the treatment in a hidden way	9 (1.1)	0.6–2.2
***Which clinician-patient communication do you mainly use to avoid nocebo-related effects?***		
Evaluate and modify patient’s anxieties, doubts and expectations	298 (37.7)	34.3–41.2
Use an empathic and authentic communication style	233 (29.5)	26.3–32.8
Provide adequate information (e.g., pathology, diagnosis, treatment, adverse events)	145 (18.3)	15.7–21.2
Investigate previous experiences of therapeutic failure	38 (4.8)	3.5–6.6
Ask the patient to summarize the information provided to avoid misunderstanding	34 (4.3)	3.0–6.0
Use images and narrative	26 (3.3)	2.2–4.8
Ask the patient to give questions	17 (2.1)	1.3–3.5

**n*, number of participants;%, percentage; 95% CI, 95% confidence interval.*

### Education of Nocebo-Related Effects

The majority of OMTs considered their education about nocebo-related effects as “medium” (*n* = 381; 48.2%; 95% CI 44.6–51.7), followed by “limited” (*n* = 218; 27.6%; 95% CI 24.5–30.8) and “very good” (*n* = 165; 20.9%; 95% CI 18.1–23.9). Some of them considered it “absent” (*n* = 20; 2.5%; 95% CI 1.6–3.9) and a few “complete” (*n* = 7; 0.9%; 95% CI 0.4–1.9).

Most participants believed that the management of nocebo-related effects should be taught in “bachelor degree” (*n* = 622; 78.6%; 95% CI 75.6–81.4). Many respondents suggested that the education should be preferably provided during a “post-graduation diploma” (*n* = 77; 9.8%; 95% CI 7.8–12.1) and some of them as “e-learning/advanced distance learning” (*n* = 55; 7.0%; 95% CI 5.3–9.0). The less chosen options were “master of science degree” (*n* = 22; 2.8%; 95% CI 1.8–4.2), “Philosophy doctor degree” (*n* = 15; 1.9%; 95% CI 1.1–3.2).

### Correlation Between Variables

Strength of association between variables was weak, with a Cramer’s V below (from 0.1 to 0.3) the established threshold (Cramer’s V < 0.5) in all correlations, such as between socio-demographic variables of section (A) and responses given in section (B) and (C) of the survey.

## Discussion

This is the first national survey that investigates the knowledge of Italian OMTs regarding nocebo-related effects and CFs. The main finding of this study suggests that OMTs are aware of the presence of nocebo-related effects in their clinical practice and that these effects can be triggered by CFs.

According to current evidences (Miller and Miller, 2015; Carlino and Benedetti, 2016), Italian OMTs defined nocebo-related effects as due to the negative psychosocial context around the therapy, composed of both internal and external elements to the patient and capable to influence his/her therapeutic outcomes through specific biological bases, thus reflecting an adequate knowledge of the topic.

Our participants identified in the associative learning and expectations the main mechanisms of action explaining nocebo-related effects. As reported in several studies, the repetitive negative associations of the therapy with CFs (e.g., specific color and shape of a medicine) (Faasse and Martin, 2018), similar and negative previous experiences (Colloca et al., 2010; Testa and Rossettini, 2016) and verbal messages highlighting negative expectations (e.g., “you will receive a medication which will increase your pain”) (Blasini et al., 2017) can trigger nocebo-related effects both in healthy people (Colloca et al., 2008; Bingel et al., 2011) and in patients (Damien et al., 2018). Instead, OMTs considered genetic as the less influent mechanism contrary to evidence that have identified the involvement of specific genes such as catechol-*O*-methyltransferase (COMT) in the development of nocebo-related effects (Wendt et al., 2014).

Italian OMTs reported that they encountered nocebo-related effects in their clinical practice, and they are convinced that these effects are triggered by specific CFs present in the therapeutic context. The most influential CFs were those mainly related to the encounter between patient and physiotherapist, that represents a fundamental moment in which biopsychosocial components are investigated, symbolizing the foundations for the therapeutic alliance in physiotherapy (Miciak et al., 2018; Moore et al., 2020). In detail, the lack of empathetic therapeutic alliance with the patient (Fuentes et al., 2014), the patient’s negative expectations as well as previous negative experience(s) (Testa and Rossettini, 2016), the physiotherapist’s negative verbal communication, attitudes and pessimistic behavior (Oliveira et al., 2012) have been all shown to negatively influence subjective (e.g., pain, anxiety) and objective (e.g., function, disability) outcomes in patient with musculoskeletal pain. Instead, the printed information about the therapy and the hidden administration of the therapy (Wand et al., 2012), the inaccurate design (Schweitzer et al., 2004), the lack of patient’s familiarity about the therapy (Faasse and Martin, 2018) and the lack of physiotherapist’s uniform (Mercer et al., 2008) have been considered less influent CFs likelihood because of a OMTs’ poor of awareness of their negative clinical importance. Overall, our findings suggest to OMTs the need to consider CFs as triggers of nocebo-related effects capable to negatively impact patients’ outcomes in accordance to the evidences reported in medicine (Colloca and Barsky, 2020) and physiotherapy (Rossettini et al., 2020).

OMTs communicated nocebo-related effects mainly during the decision of the therapeutic plan, balancing the positive features of the treatment with the negative ones. According to evidence available, no information should be omitted during the discussion of the positive and negative effects of treatment, offering to clinicians three options of communication: (a) explaining and highlighting as first the desired positive treatment effects (Kleine-Borgmann and Bingel, 2018); (b) reframing the information on negative effects in a positive way (Enck et al., 2013; Bingel, 2014); and (c) informing patients about the presence of nocebo-related effects and their relevance in the treatment (Crichton and Petrie, 2015). Only few OMTs did not inform their patients, resulting in a non-transparent and deceptive communication that threatens the respect of ethical principles behind the therapy administration (e.g., the principle of autonomy, the informed consent) (Colloca and Finniss, 2012; Klinger et al., 2017).

Italian OMTs referred they managed nocebo-related effects by teaching and training patient’s strategies to control unintended negative effects of treatment. Orthopedic manual therapists also reported having adopted an empathic and authentic communication style aimed to evaluate and modify patients’ anxieties, doubts and expectations. Overall, these results highlight how an adequate interaction between the clinician and the patient is essential to minimize nocebo-related effects (Dieppe et al., 2016; Blasini et al., 2018), underlining the importance of education (Wijma et al., 2016; Hoon et al., 2017). During the therapeutic encounter, it has been shown that an appropriate education can influence the outcome (e.g., pain, function) (Louw et al., 2016) and improve the patients’ self-efficacy (Jönsson et al., 2018) in patients with musculoskeletal pain.

Most OMTs considered to have a medium level of education about nocebo-related effects suggesting that this topic should be taught mainly during the bachelor degree, as suggested internationally (Evers et al., 2018) and previously reported among clinicians (Rossettini et al., 2018b; Palese et al., 2019) and students (Cadorin et al., 2020) in physiotherapy and in nursing field.

### Strengths and Weakness of the Study

The current survey presented some strengths. A high response rate was achieved (61.4%), confirming the willingness of OMTs to participate in this study. Moreover, authors have adopted an online survey to understand the opinion of the target population. The methodological choice was previously used in surveys on placebo-related effects and CFs representing a valid tool aimed to capture the perspective of a large sample of healthcare providers (Rossettini et al., 2018b).

As a weakness, a group of Italian physiotherapists specialized in OMT educated mostly in managing musculoskeletal pain in the private healthcare settings (AIFI, 2020) were involved. Therefore, their response can be not generalizable to non-specialized physiotherapists (e.g., not OMTs), working in other fields (e.g., neurology) and employed in different settings (e.g., hospital), thus suggesting future studies in this field. Most of participants worked full-time, in the North of Italy and for less than 10 years: these are all factors that could have influenced beliefs and knowledge regarding nocebo effects, limiting the generalizability of findings (Rossettini et al., 2018b). Moreover, data were self-reported introducing a social and recall bias that can have affected the findings. Furthermore, the format of asking participants how to define nocebo effects with a closed question with three options could have given some prior cues to the participant, thus biasing their reported knowledge on the topic. Finally, despite the anonymity was guaranteed some participants might have misreported some data (Eysenbach, 2004; Palese et al., 2019).

## Conclusion

In summary this national survey shows that OMTs are aware of the presence of nocebo-related effects in their clinical practice and that these effects can be triggered by CFs. From a policymakers’ perspective, it is recommended to ensure an appropriate knowledge on nocebo-related effects among healthcare providers aimed at minimizing their negative impact in clinical practice, including this topic in undergraduate education.

## Data Availability Statement

The raw data supporting the conclusions of this article will be made available by the authors, without undue reservation.

## Ethics Statement

The studies involving human participants were reviewed and approved by Liguria Clinical Experimental Ethics Committee (P.R.236REG2016, accepted on 19/07/2016). The patients/participants provided their written informed consent to participate in this study.

## Author Contributions

GR, TG, and MT were responsible for project administration. GR, TG, and AP were major contributors in writing (original draft). GR and MT were responsible for the resources. AP, LC, MF, and AT contributed to the investigation. GR, TG, AP, CM, MMi, AT, MMa helped in data curation and formal analysis. LC, MF, AT, MMi, MMa, and MT contributed to the writing (review and editing). All authors read and approved the final version of the manuscript.

## Conflict of Interest

GR leads education programs on placebo, nocebo effects and contextual factors in healthcare to under- and post-graduate students along with private CPD courses. The remaining authors declare that the research was conducted in the absence of any commercial or financial relationships that could be construed as a potential conflict of interest.

## Footnotes

1 www.surveymonkey.com
