# Supplementary material for: What Physiotherapists Specialized in Orthopedic Manual Therapy Know About Nocebo-Related Effects and Contextual Factors: Findings From a National Survey
Source: Front Psychol. 2020 Oct 20;11:582174. doi: 10.3389/fpsyg.2020.582174 (PMC7606996; doi:10.3389/fpsyg.2020.582174)
Supplement: Supplementary file 1 [file Table_1.DOCX]

**Supplementary File 1: Italian version of the questionnaire**

**Benvenuto!**

Gentile collega grazie per prendere parte a questa survey.

Questa indagine serve a chiarire il ruolo degli effetti nocebo nell’influenzare il risultato terapeutico nell’attività clinica fisioterapica.

Gli effetti nocebo rappresentano “gli effetti avversi non specifici generati del contesto psicosociale negativo che ruota attorno alla terapia”.

Consideriamo importante studiarli nella pratica clinica quotidiana del fisioterapista perché gli effetti nocebo determinano una riduzione dell’efficacia e dell’efficienza della terapia, favorendone la non aderenza.

Gentilmente rispondi alle seguenti domande sulla base della tua personale esperienza e pratica clinica.

La compilazione dell’intero questionario è volontaria e richiede poco più di 5 minuti. Le risposte date sono completamente anonime e saranno utilizzate solamente per gli scopi di questa ricerca.

Cliccando sul link del questionario, tu fornisci il tuo consenso a partecipare allo studio. Quando completi la pagina, clicca su “Prosegui” per salvare le tue risposte. Se decidi di abbandonare il questionario, seleziona “Uscita”.

**Caratteristiche sociodemografiche**

***Quale è il tuo sesso?*** *[Seleziona]*

- Maschio
- Femmina

***Quanti anni hai?*** *[Completa numericamente es. 34]*

……….

***Da quanti anni sei fisioterapista?*** *[Completa numericamente es. 10]*

……….

***In che zona di Italia lavori?*** *[Seleziona]*

- Nord
- Centro
- Sud

***In che settore operi?*** *[Seleziona]*

- Pubblico
- Privato

***Quale è il tuo inquadramento professionale?*** *[Seleziona]*

- Dipendente
- Libero professionista

***Quale è il tuo ambito lavorativo?*** *[Seleziona]*

- Ambulatorio
- Ospedale
- Strutture assistenziali (casa di riposo, RSA)

***Che tipologia di pazienti riabiliti prevalentemente?*** *[Seleziona]*

- Pediatrici (< 18 anni)
- Adulti (18-65 anni)
- Anziani (> 65 anni)

***Quale è il tuo campo di intervento prevalente?*** *[Seleziona]*

- Muscoloscheletrico
- Neurologico
- Oncologico
- Cardio-respiratorio
- Uro-ginecologico

***Quante ore lavori a settimana?*** *[Seleziona]*

- 1-15
- 16-30
- 31-45
- 46-60
- più di 60 ore

**Frequenza degli effetti nocebo**

***Con che frequenza hai riscontrato effetti nocebo nella tua carriera clinica?*** *[Seleziona]*

- Sempre (100%)
- Spesso (75%)
- A volte (50%)
- Raramente (25%)
- Mai (0%)

**Credenze riguardo la capacità dei fattori di contesto di scatenare effetti nocebo**

***Quanto credi che gli effetti nocebo possano essere scatenati dai seguenti fattori presenti nel contesto terapeutico?*** *[Seleziona]*

|  | Moltissimo | Molto | Abbastanza | Poco | Per nulla |
| --- | --- | --- | --- | --- | --- |
| -La scarsa reputazione del fisioterapista (es. esperienza, sicurezza, qualifica) |  |  |  |  |  |
| -La mancanza della divisa del fisioterapista (es. camice, casacca) |  |  |  |  |  |
| -I comportamenti e le attitudini non ottimiste (es. verso la patologia) |  |  |  |  |  |
| -Le aspettative negative del paziente (es. verso la terapia) |  |  |  |  |  |
| -Le precedenti esperienze negative del paziente (es. verso la terapia) |  |  |  |  |  |
| -La comunicazione verbale negativa da parte del fisioterapista (es. linguaggio medico, mancanza di istruzioni positive associate alla terapia) |  |  |  |  |  |
| -La comunicazione non verbale negativa da parte del fisioterapista (es. postura in chiusura, gesti, assenza di contatto oculare, espressioni facciali) |  |  |  |  |  |
| -La mancanza di alleanza terapeutica empatica (es. ascolto non attivo) |  |  |  |  |  |
| -Le informazioni sulla terapia di altri pazienti (es. comunicazione o osservazione di risposte negative) |  |  |  |  |  |
| -Le informazioni sulla terapia cartacee (es. bugiardino dei farmaci) |  |  |  |  |  |
| -Le informazioni sulla terapia dei media (es. internet, social, telegiornali) |  |  |  |  |  |

| -La somministrazione nascosta della terapia da parte del fisioterapista (es. impossibilità di vedere quando viene erogata la terapia) |  |  |  |  |  |
| --- | --- | --- | --- | --- | --- |
| -L’interruzione improvvisa della terapia da parte del fisioterapista |  |  |  |  |  |
| -Il marketing della terapia (es. costo, marca, colore e la forma) |  |  |  |  |  |
| -La mancanza di familiarità del paziente verso la terapia (es. terapia nuova) |  |  |  |  |  |
| -La mancanza di un approccio centrato sul paziente (es. l’impossibilità di scegliere la terapia in maniera condivisa con il clinico) |  |  |  |  |  |
| -Il contatto fisico non adeguato con il fisioterapista (es. invasività) |  |  |  |  |  |
| -La mancanza di un ambiente confortevole (es. non idonea illuminazione, luce, temperatura) |  |  |  |  |  |
| -L’architettura sanitaria non adeguata (es. non idonei punti luce, indicazioni) |  |  |  |  |  |
| -Il design ambientale non accurato (es. assenza di decorazioni, ornamenti, colori) |  |  |  |  |  |

**Comunicazione degli effetti nocebo**

***Come comunichi al paziente prevalentemente gli effetti nocebo?*** *[Seleziona]*

- Non dici nulla
- Minimizzi le informazioni negative sugli eventi avversi evitando di riportare tutti gli elementi
- Bilanci gli aspetti positivi della terapia con quelli negativi
- Spieghi attentamente gli effetti ed il ruolo giocato dal contesto negativo

***Quando comunichi al paziente prevalentemente gli effetti nocebo?*** *[Seleziona]*

- Non lo comunichi
- Durante l’anamnesi
- Durante l’esame clinico
- Durante la formulazione della diagnosi
- Durante la stesura del piano terapeutico
- Durante la somministrazione della terapia

**Meccanismi di azione degli effetti nocebo**

***Quali meccanismi d’azione possono spiegare secondo la tua esperienza/competenza prevalentemente gli effetti nocebo?*** *[Seleziona]*

- Aspettativa del paziente
- Apprendimento associativo (condizionamento)
- Apprendimento sociale
- Precedenti esperienze
- Tratti psicologici
- Neurofisiologici
- Genetica

**Gestione degli effetti nocebo**

***Quale dei seguenti interventi adotti prevalentemente per evitare gli effetti nocebo?*** *[Seleziona quello che utilizzi maggiormente]*

- Non fai nulla
- Presenti prima gli aspetti positivi della terapia e poi quelli avversi
- Spieghi gli effetti nocebo usando metodi illustrativi (es. video, figure, grafici e percentuali) e un linguaggio semplice
- Rimandi a informazioni evidence-based presenti in internet
- Insegni ed alleni le strategie nel paziente per gestire gli effetti nocebo
- Ottimizzi le aspettative verso il trattamento e gli effetti nocebo
- Utilizzi pre-trattamenti con una ridotta percentuale di effetti nocebo (es. trattamenti di prova attivi o inerti)
- Adotti, in maniera nascosta, una riduzione graduale del trattamento

***Quale comunicazione clinico-paziente adotti prevalentemente per evitare gli effetti nocebo?*** *[Seleziona quello che utilizzi maggiormente]*

- Utilizzi uno stile comunicativo empatico ed autentico
- Fornisci adeguate informazioni (es. patologia, diagnosi, trattamento e effetti avversi)
- Utilizzi immagini e narrativa
- Richiedi al paziente di riassumere le informazioni fornite per evitare mal interpretazioni
- Richiedi al paziente di fare domande
- Valuti e correggi le ansie, i dubbi e le aspettative del paziente
- Indaghi precedenti esperienze di fallimento terapeutico

**Formazione sugli effetti nocebo**

***Come reputi la tua formazione attuale sugli effetti nocebo?*** *[Seleziona]*

- Ottima (100%)
- Buona (75%)
- Sufficiente (50%)
- Scarsa (25%)
- Assente (0%)

***In quale fase del percorso formativo dovrebbe essere insegnata la gestione degli effetti nocebo?*** *[Seleziona]*

- Laurea di primo livello
- Master universitario
- Laurea magistrale
- Dottorato di ricerca
- E-learning/a distanza

**Definizione di effetti nocebo**

***Come definiresti, alla luce di questa indagine, gli effetti nocebo?*** *[Seleziona]*

- Gli effetti di una procedura sanitaria in grado di creare aspettative negative (es. somministrare una sostanza attiva o inerte associando espressioni verbali di peggioramento dei sintomi)
- Gli effetti del contesto psicosociale attorno alla terapia e al paziente con specifiche basi biologiche (es. indipendentemente dalla sostanza somministrata, vera o inerte, associare parole ed altri elementi dell’incontro terapeutico che determinano un peggioramento dei sintomi)
- Le risposte avverse osservate nei soggetti del gruppo di controllo di trial clinici randomizzati (es. risposte avverse generate dalla lettura delle possibili conseguenze della terapia, riportate nel consenso informato)
