# Supplementary material for: What Physiotherapists Specialized in Orthopedic Manual Therapy Know About Nocebo-Related Effects and Contextual Factors: Findings From a National Survey
Source: Front Psychol. 2020 Oct 20;11:582174. doi: 10.3389/fpsyg.2020.582174 (PMC7606996; doi:10.3389/fpsyg.2020.582174)
Supplement: Supplementary file 2 [file Table_2.DOCX]

**Supplementary File 2: English version of the questionnaire**

**Welcome to this survey!**

Dear colleague,

Thank you for taking part in this survey. This survey aims to clarify the role of nocebo-related effects in influencing the therapeutic outcome of physiotherapy’s clinical practice.

Nocebo-related effects represent the “nonspecific adverse events caused by the psychosocial negative context around the therapy”.

We consider important to study them in the daily physiotherapist’s clinical practice because nocebo-related effects determine an efficacy and efficiency reduction of the therapy, encouraging non-adherence.

Please answer the following questions based on your personal clinical experience. Completion of the entire questionnaire is voluntary and will take you just over 5 minutes. Your answers are completely anonymous and will only be used for the purposes of this research.

By clicking on the link to the survey, you provide your consent to participate in the study. Whenever you complete the page, click on "Next" to save your answer. If you decide to abandon the survey, click on "Exit".

**Socio-demographic characteristics**

***What is your gender?*** *[select]*

- Male
- Female

***How old are you?*** *[complete numerically e.g., 34]*

……….

***How long have you been licensed as a physiotherapist?*** *[complete numerically e.g., 10]*

……….

***Which part of Italy do you work in?*** *[select]*

- North
- Centre
- South

***Which is your clinical workplace?*** *[select]*

- Public health care setting
- Private health care setting

***Which is your type of work?*** *[select]*

- Employee
- Freelance professional

***Which is your setting?*** *[select]*

- Outpatient clinic
- Hospital
- Residential care (nursing home, RSA)

***Which kind of patients do you mainly treat?*** *[select]*

- Paediatrics (< 18 years old)
- Adults (18-65 years old)
- Older people (>65 years old)

***What is your main field of intervention?*** *[select]*

- Musculoskeletal
- Neurological
- Oncological
- Cardiorespiratory
- Uro-gynecological

***How many hours do you work each week?*** *[select]*

- 1-15
- 16-30
- 31-45
- 46-60
- > 60

**Frequency of nocebo-related effects**

***How often in your career have you found nocebo-related effects?*** *[select]*

- Always (100%)
- Often (75%)
- Sometimes (50%)
- Rarely (25%)
- Never (0%)

**Beliefs of contextual factors as triggers of nocebo-related effects**

***How much do you believe that nocebo-related effects can be triggered by the following factors of the therapeutic context?*** *[select]*

|  | **A lot of** | **Much** | **Enough** | **Few** | **Not at all** |
| --- | --- | --- | --- | --- | --- |
| -Weak professional reputation (e.g., qualification, expertise of physiotherapist)  -Lack of uniform (e.g., white coat of physiotherapist)  -Negative attitudes and pessimistic behaviour (e.g., towards a patient’s dysfunctions)  -Patient’s negative expectation (e.g., towards a physiotherapy treatment)  -Patient’s previous negative experience (e.g., towards a physiotherapy treatment)  -Negative verbal communication (e.g., medical language, lack of positive messages associated with the treatment)  -Negative non-verbal communication (e.g., closing posture, gestures, absence of eye contact, facial expressions)  -Lack of empathetic therapeutic alliance with the patient (e.g., lack of active listening)  -Information about the therapy delivered by other patients (e.g., negative communicated or observed responses)  -Printed information about the therapy (e.g., medical leaflets)  -Information about the therapy from the media (e.g., internet, social media, television news)  -Hidden therapy (e.g., impossibility for the patient to see when the therapy is delivered)  -Sudden interruption of the therapy (e.g., to attend other patients or colleagues)  -Marketing of the therapy (e.g., cost, brand, colour, shape)  -Lack of patient’s familiarity with the therapy (e.g., new therapy)  -Lack of patient-centred approach (e.g., not shared-decision of physiotherapy treatment)  -Inappropriate physical contact with the patient (e.g., invasiveness of touch)  -Lack of comfortable setting (e.g., inappropriate lighting, temperature)  -Inadequate environmental architecture (e.g., inappropriate highlights, indicators)  -Inaccurate design (e.g., absence of decorations, ornaments, colours) |  |  |  |  |  |
|  |  |  |  |  |  |
|  |  |  |  |  |  |
|  |  |  |  |  |  |
|  |  |  |  |  |  |
|  |  |  |  |  |  |
|  |  |  |  |  |  |
|  |  |  |  |  |  |
|  |  |  |  |  |  |
|  |  |  |  |  |  |

**Communication of nocebo-related effects**

***How do you mainly communicate nocebo-related effects to the patient?*** *[select]*

- Do not say anything
- Minimize negative information on nocebo-related effects by not reporting all the elements
- Balance the positive features of the treatment with the negative ones
- Carefully explain the effects and the role played by the negative context

***When do you mainly communicate nocebo-related effects to the patient?*** *[select]*

- Do not communicate anything
- During the anamnesis
- During the clinical examination
- During the formulation of the diagnosis
- During the decision of the therapeutic plan
- During the administration of the treatment

**Mechanisms of action of nocebo-related effects**

***Which mechanisms of action, based on your experience/competence, can mainly explain nocebo-related effects?*** *[select]*

- Patient’s expectation
- Associative learning (e.g., conditioning)
- Social learning
- Previous experiences
- Psychological traits
- Neurophysiological
- Genetic

**Management of nocebo-related effects**

***Which interventions do you mainly use to avoid nocebo-related effects?*** *[select the one that you mainly use]*

- Do not do anything
- Present first the positive features of the treatment and then the negative ones
- Explain nocebo-related effects using illustrative methods (e.g., videos, figures, graphs and percentages) and simple language
- Refer to evidence-based information on the Internet
- Teach and train patient’s strategies to manage nocebo-related effects
- Optimize expectations towards treatment and nocebo-related effects
- Use pre-treatments with a reduced percentage of nocebo-related effects (e.g., active or inert treatment-test)
- Adopt a gradual reduction of the treatment in a hidden way

***Which clinician-patient communication do you mainly use to avoid nocebo-related effects?*** *[select the one that you mainly use]*

- Use an empathic and authentic communication style
- Provide adequate information (e.g., pathology, diagnosis, treatment, adverse events)
- Use images and narrative
- Ask the patient to summarize the information provided to avoid misunderstanding
- Ask the patient to give questions
- Evaluate and modify patient’s anxieties, doubts and expectations
- Investigate previous experiences of therapeutic failure

**Education on nocebo-related effects**

***How do you consider your current education about nocebo-related effects?*** *[select]*

- Complete (100%)
- Very good (75%)
- Medium (50%)
- Limited (25%)
- Absent (0%)

***In which educational course should management of nocebo-related effects be taught?*** *[select]*

- Bachelor degree
- Post-graduation diploma
- Master of science degree
- Philosophical doctor degree (PhD)
- E-learning/advanced distance learning

**Definition of nocebo-related effects**

***How would you define, in the light of this survey, nocebo-related effects?*** *[select]*

- Health procedure effects able to create negative expectations (e.g., administering an active or inert substance by combining verbal expressions of worsening symptoms)
- Psychosocial effects of the context around therapy and patient with specific biological bases (e.g., associate words and other therapeutic encounter’s elements that cause a worsening of symptoms, regardless the real or inert administered substance)
- Adverse responses observed in people of the control group of randomized clinical trials (e.g., adverse responses caused by reading, in the informed consent, the possible consequences of the therapy)
